# ATRI EDC: a novel cloud-native remote data capture system for large multicenter Alzheimer’s disease and Alzheimer’s disease-related dementias clinical trails

**DOI:** 10.1093/jamiaopen/ooab119

**Published:** 2022-01-17

**Authors:** Gustavo A Jimenez-Maggiora, Stefania Bruschi, Hongmei Qiu, Jia-Shing So, Paul S Aisen

**Affiliations:** Alzheimer’s Therapeutic Research Institute, Department of Neurology, Keck School of Medicine of USC, Los Angeles, California, USA

**Keywords:** data management, clinical trials, Alzheimer disease, clinical research informatics, remote electronic data capture

## Abstract

**Objective:**

The Alzheimer’s Therapeutic Research Institute (ATRI) developed a novel clinical data management system, the ATRI electronic data capture system (ATRI EDC), to address the complex regulatory, operational, and data requirements that arise in the conduct of multicenter Alzheimer’s disease and Alzheimer’s disease-related dementias (AD/ADRDs) clinical trials. We describe the system, its utility, and the broader implications for the field of clinical trials and clinical research informatics.

**Materials and Methods:**

The ATRI EDC system was developed, tested, and validated using community-based agile software development methods and cloud-native single-page application design principles. It offers an increasing number of application modules, supports a high degree of study-specific configuration, and empowers study teams to effectively communicate and collaborate on the accurate and timely completion of study activities.

**Results:**

To date, the ATRI EDC system supports 10 clinical studies, collecting study data for 4596 participants. Three case descriptions further illustrate how the system’s capabilities support diverse study-specific requirements.

**Discussion:**

The ATRI EDC system has several advantages: its modular capabilities can accommodate rapidly evolving research designs and technologies; its community-based agile development approach and community-friendly licensing model encourage collaboration per the principles of open science; finally, with continued development and community building efforts, the system has the potential to facilitate the effective conduct of clinical studies beyond the field of AD/ADRD.

**Conclusion:**

By effectively addressing the requirements of multicenter AD/ADRD studies, the ATRI EDC system supports ATRI’s scientific mission of rigorously testing new AD/ADRD therapies and facilitating the effective conduct of multicenter clinical studies.

## Lay Summary

The Alzheimer’s Therapeutic Research Institute (ATRI) developed a novel clinical data management system, the ATRI electronic data capture system, to address the complex regulatory, operational, and data requirements that arise in the conduct of multicenter Alzheimer’s disease and Alzheimer’s disease-related dementias clinical trials. We describe the system, its utility, and the broader implications for the field of clinical trials and clinical research informatics.

## INTRODUCTION

Alzheimer’s disease (AD) and Alzheimer’s disease-related dementias (ADRDs) represent a serious public health crisis for healthcare systems around the world.^1^ Despite significant challenges in the development of new disease-modifying therapies (DMTs) for AD/ADRD, the pipeline of candidate therapeutics remains substantial.^2^ Considering these development efforts, the need for dedicated infrastructure and expertise to perform rigorous and efficient testing of candidate therapies became evident. The Alzheimer’s Therapeutic Research Institute (ATRI) (https://keck.usc.edu/atri/) was founded in 2015 to address this need. As part of the Keck School of Medicine of USC, ATRI has grown into the largest NIH-funded AD/ADRD clinical trial coordinating center (CTCC) in the United States, designing, conducting, and analyzing over 20 multicenter clinical trials since its founding. As a comprehensive CTCC, ATRI provides leadership and expertise in multiple clinical research specialties and robust infrastructure. A core component of this infrastructure is an effective research data management platform. This platform, which must adapt to study-specific requirements, is anchored by a web-based clinical data management system (CDMS) developed by ATRI: the ATRI electronic remote data capture system (ATRI EDC). The purpose of this article is to describe the ATRI EDC system, its utility, and the broader implications for the field of clinical trials and clinical research informatics.

### AD/ADRD clinical trial requirements

#### Operational complexity

Multicenter AD/ADRD clinical trials are large, complex research enterprises that require coordination across multiple stakeholders—public and private sponsors, regulators, study teams, vendors, and trial sites. In this setting, timely communication and collaboration are critical to achieving study objectives and milestones effectively. Gaps can introduce compliance issues, reduce data quality, and compromise participant safety.

#### Geographic complexity

Multicenter AD/ADRD clinical trials are increasingly global in scope. Several clinical trials managed by ATRI, such as the Anti-Amyloid Treatment in Asymptomatic Alzheimer’s study (A4 study),^3^ are conducted at trial sites across Asia, Australia, Europe, and North America. Multinational settings introduce a complex patchwork of regulatory, operational, data security, and data privacy requirements.

#### Complex data types, data flows, and study designs

AD/ADRD clinical trials require the management of large, heterogeneous databases composed of structured, unstructured, binary, and complex data types. These data types include clinical, biosamples (fluid, tissue), neuroimaging, genetic, multimedia (audio, video), and study documentation. The sources of data are increasingly specialized and disparate, requiring ongoing monitoring of data quality and integrity. Mappings across databases must be established and maintained. Novel data types, such as sensor (IoT), wearable, administrative, log, and geospatial data, must also be managed and mapped with existing databases.^4^ The increased adoption of adaptive methods and platform designs in AD/ADRD trials introduces more demanding operational and data quality requirements.^5^^,^^6^ Adaptive designs require systems that are highly available and flexible to effectively assess data quality and maintain timely data flows to support interim analyses yielding adjustments to participant allocation and the number of treatment arms.

#### Extended study follow-up periods

Due to uncertain rates of disease progression, AD/ADRD clinical trial designs typically require extended follow-up periods of 18 months or more. Add to this protracted enrollment periods that span several years, and clinical trial durations frequently extend 5 years or more; instances of almost decade-long AD/ADRD clinical trials are not uncommon. Long-duration study designs present unique requirements that necessitate sophisticated forecasting and risk-based planning. In this context, sustainability, scalability, and flexibility in the face of unforeseen contingencies are critical.

#### Challenges to data quality

AD/ADRD trials are notoriously labor-intensive and expensive to conduct due to complex procedures, high screen failure rates, and prolonged follow-up periods.^7^ The shift from intervention to prevention studies, which rely on the timely assessment of genetic, neuroimaging, and biofluid biomarkers, further exacerbate these challenges.^8^^,^^9^ Consequently, it is common for AD/ADRD trials to experience high site staff turnover and low-enrolling sites, potentially leading to data quality issues.

### Assessment of existing clinical data management systems

In late 2015, ATRI conducted an initial assessment of several open-source and commercial CMDSs against the previously stated requirements.^10–14^ In each case, the systems offered limited capabilities, inflexible licensing models, prohibitive costs, or were no longer under active development. Therefore, ATRI decided to develop a novel CDMS (ATRI EDC) using cloud-native architecture and modern application development tools to address these requirements.

## MATERIALS AND METHODS

### ATRI EDC overview

The ATRI EDC system was designed to facilitate the conduct of multicenter AD/ADRD clinical studies by providing a scalable, extensible, cost-effective, and regulatory-compliant electronic remote data capture (eRDC) platform to manage large, heterogeneous data types and model complex dataflows and workflows. The system provides study teams with real-time visibility into a study’s operations and facilitates communication and collaboration between the CTCC, sponsors, vendors, and sites. It accomplishes this by providing user interfaces that leverage contemporary web application design practices and methods (Table 1). At its core, the system uses metadata derived from a study’s protocol (schedule of events, case report forms, data types, and key outcomes) to present a structured, study-specific, web-based clinical data management environment that guides users through the accurate and timely completion of study activities.

**Table 1.** Technologies used to develop and host the ATRI EDC system

Domain	Subdomain	Tool/service name
Applications/tools	Source code management	GitHub
	Client-side web application development	AngularJS
		ReactJS
	Project management	Atlassian Suite—JIRA, Confluence
	Continuous integration (CI), continuous deployment (CD)	Travis CI
	Code test coverage analysis	Coveralls
	Server-side web application development	Django—Python Web Framework
	Application programming interface (API) development	Django REST Framework
	Web application testing	Selenium
	Identity management framework	SAML SSO
Cloud-based IT infrastructure—Amazon Web Services (AWS)	Compute	EC2
	Batch compute	AWS Batch
	Serverless compute	AWS Lambda
	Web application framework	Amazon Elastic Beanstalk (EB)
	Containers	Amazon Elastic Container Service (ECS)
	Email	Amazon Simple Email Service (SES)
	Database	DynamoDB
		Amazon RDS
	Machine learning	Amazon Sagemaker
	Machine learning-based code analysis	Amazon CodeGuru
	Developer tools	AWS CodePipeline
		AWS CodeBuild
	Governance and management	Amazon CloudWatch
		AWS CloudTrail
		AWS Config
		AWS CloudFormation
		Amazon Macie
		AWS System Manager
		AWS Trusted Advisor
	Networking and content delivery	Amazon VPC
		Amazon CloudFront
		AWS API Gateway
		Amazon Route 53
	Security, identity, and compliance	AWS Certificate Manager
		AWS IAM
		AWS GuardDuty
		AWS Secrets Manager
		AWS Key Management Service (KMS)
	Storage	Amazon S3
	Application integration	Amazon Step Functions
		Amazon Simple Queue Service (SQS)
		Amazon Simple Notification Service (SNS)
Analytics and statistical reporting	Scientific and statistical computing	Python
		R
	Website analytics	Google Analytics
Support	User support	Teamwork Desk
	Technical support	FreshService

*Note:* Some tools and services are listed more than once due to their application in multiple domains.

### System development

#### Community-based agile development process

The initial version of the ATRI EDC system was released in September 2016, after 10 months of development. The development process adhered to the system development life cycle (SDLC) model, adapted to incorporate rapid prototyping and iterative stakeholder assessment consistent with Agile software development principles and methods.^15^^,^^16^ During this process, the development team met with multidisciplinary stakeholders frequently to present system prototypes and elicit feedback. The SDLC incorporated test-driven development, behavior-driven development and testing, continuous integration, and continuous deployment methods to build, test, and validate the system continuously (Table 1).

#### Web application design principles

The ATRI EDC system was built using open-source web application frameworks and tools (Table 1) following the single-page application design pattern.^17^ This approach aims to provide users with a responsive, fluid experience reminiscent of a desktop application via a browser-based application. A complete description of the single-page application design pattern is beyond the scope of this article.

#### Partnership with cloud IT provider

During the development process, Amazon Web Services (AWS)^18^ was selected as the preferred cloud platform for the ATRI EDC project. This choice led to the establishment of an extensive ongoing partnership between ATRI and AWS that provides access to expertise, training, and support in cloud-based architecture and best practices. While designed to be platform-agnostic, the ATRI EDC system’s performance characteristics can be optimized when running on the AWS cloud (Table 1). This optimized configuration leverages specific AWS services and features to achieve increased performance, security, scalability, and resilience.

#### Regulatory compliance

In the United States, computer systems used to manage data for submissions to regulatory agencies must comply with 21 CRF Part 11 (Part 11).^19^ This requirement applies to the ATRI EDC system. Traditional methods to demonstrate compliance require protracted, labor-intensive manual testing and validation procedures. These methods are costly, error-prone, lack reproducibility, and, ultimately, slow system innovation. The ATRI Informatics team developed novel automated testing and validation methods to address these challenges.^20^ Integrating these methods into the SDLC process generated efficiencies and ensured Part 11 compliance on an ongoing basis. An independent assessment of the ATRI EDC system completed in January 2018 provided further evidence of Part 11 compliance.

#### Focus on data security and data privacy

**Figure 1.**
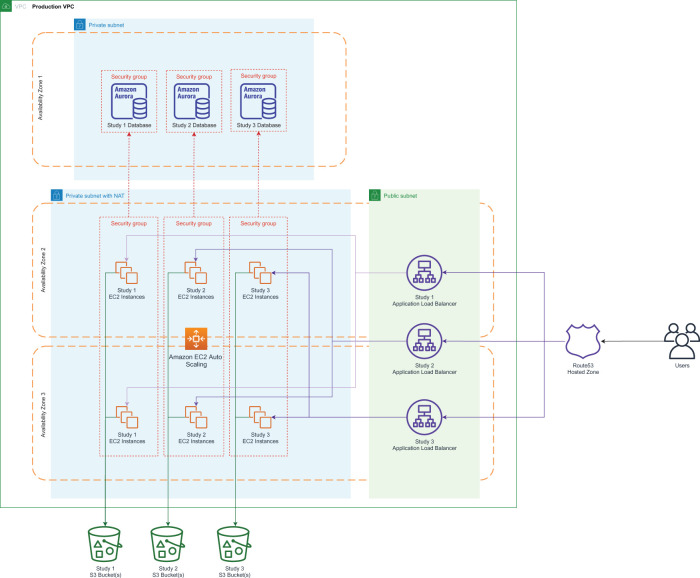
ATRI EDC AWS architecture. This cloud-based IT architecture assures strict isolation of configurations, data, and study-specific code between studies. It is implemented using a multi-account strategy that compartmentalizes core functions (production, staging and development workloads, audit, analytics, and logs) to improve security by minimizing the impact of a potential breach. Security groups and policies enforce network security, strong encryption at rest and in transit, and multifactor authentication for administrative accounts. High durability and availability are ensured via multi-region data replication and auto-scaling application groups. IT infrastructure is managed programmatically to minimize the possibility of human error in configuration management. VPC: virtual private network

Data security and data privacy are critical requirements for CDMSs. In the United States, the Health Insurance Portability and Accountability Act^21^ mandates these requirements and defines penalties for non-compliance. More stringent data regulations are in force in jurisdictions outside the United States. To address these requirements, ATRI partnered with Amazon Web Services Professional Services (AWS ProServ) to establish a HIPAA-compliant IT infrastructure to host the ATRI EDC system (Figure 1). This environment implements automated compliance and security policies using DevSecOps methods such as automated data classification, configuration, threat detection, and web application firewalls.^22^^,^^23^ As of August 2021, further work with AWS ProServ focused on compliance with HITRUST Common Security Framework^24^ and the European Union General Data Protection Regulation^25^ was underway.

### System design

#### Clinical data management system and applications

**Figure 2.**
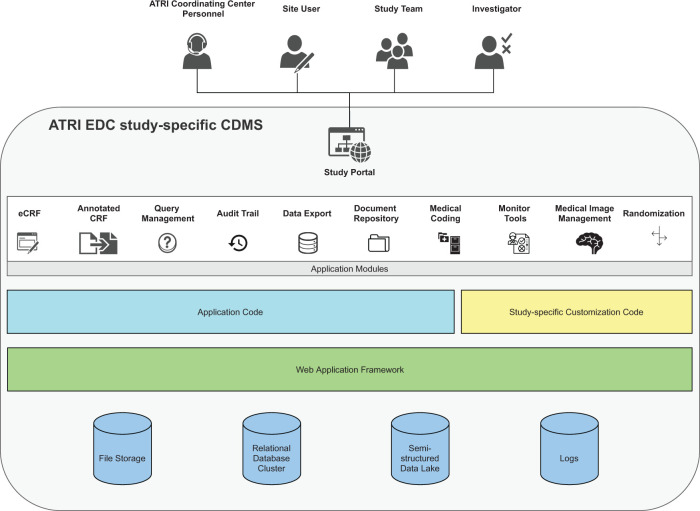
ATRI EDC study-CDMS architecture. Each study-specific CDMS is built using a dedicated set of application components hosted on shared cloud-based IT infrastructure. This design guarantees strict isolation of configurations, data, and study-specific code between studies. It also allows IT resources (compute, service, storage) to be optimized to address study-specific regulatory, operational, data, and fiscal requirements. From the user perspective, this design offers authorized users a single point of access to multiple study-specific application modules. Access to these modules is governed by role-based permissions. This approach facilitates communication and collaboration in the accurate and timely completion of study activities.

The ATRI EDC system is a general CDMS package that serves as a framework for managing independent study-specific CDMS instances (study-CDMS) (Figure 2). In this approach, each study-CDMS is customized using study-specific configurations, data types, workflows, and functionality. This approach ensures strict isolation between study-CDMSs while still allowing the use of shared IT infrastructure. Furthermore, this approach supports the implementation of good clinical data management practices, such as the use of distinct study-specific production and development study-CDMSs to support well-controlled data management processes.

#### Study team-centered management

The ATRI EDC system supports a study team-centered approach to the configuration and administration of study-CDMSs. In this approach, study teams use web-based administrative user interfaces to manage the system’s features and functionality. This approach empowers study teams and minimizes the need for IT team involvement. The use of these interfaces requires no programming skills. Configuration metadata may also be imported using spreadsheets. These spreadsheets support collaborative configuration efforts.

#### Study portal and modules

A study-CDMS’s functionality, features, and data are available via a secure study-specific web portal. Portals are assigned study-specific web addresses (URLs) to facilitate user access. Within the portal, users can easily find modules that support various study activities such as participant registration, eCRF capture, query resolution, study document retrieval, and neuroimaging study upload (Figure 2). Access rights to each module are governed by a user’s role in the study.

#### Data model and objects

**Figure 3.**
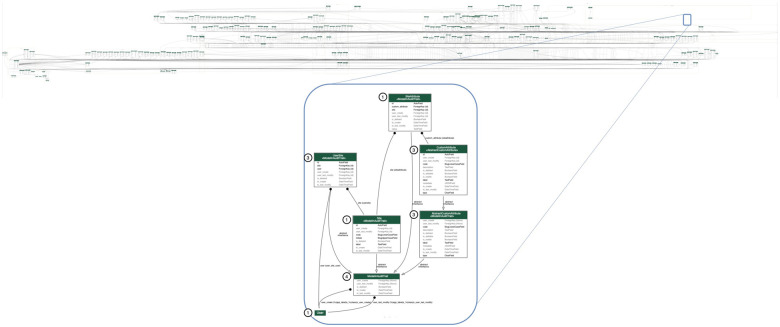
ATRI EDC entity relationship diagram (ERD). The ATRI EDC ERD visualizes the system’s data model. The data model contains over 100 entities that serve as the foundation for the system’s database. Each entity may be classified into 1 of 4 types: (1) data ledger, (2) data, (3) metadata, and (4) audit trail or history. Entities may also be divided into core and application-specific entities. To further illustrate this design, the inset diagram presents the ERD for a specific application. This application-level ERD contains 3 entity types and demonstrates the relationships between them.

The ATRI EDC system’s data model combines a data ledger and a metadata database that provide a comprehensive, immutable, and cryptographically verifiable audit trail (Figure 3). Layered atop the data ledger, the metadata model defines an elementary set of primary data types: study, participant, event, field, form, file. The primary data types may be combined or extended to define derived composite data types. Data type definitions offer a mechanism to describe data in rich detail and may include validation rules, mappings to external ontologies, and relationships with other data types. The system uses these data type definitions during data collection activities to create, correlate, and validate data object instances (DOIs) in the data ledger. Each DOI represents a unique, fully characterized, version-controlled, cryptographically signed data value. A data record, which itself is a DOI, is composed of a prespecified set of data item DOIs. The DOI construct serves multiple purposes: identification, attribution, authentication, correlation, and verification. At any point in time, the authenticity and integrity of a DOI can be verified by reviewing the ATRI EDC system’s audit trail. This data model design offers the necessary rigor and flexibility to manage the large heterogeneous data types common in AD/ADRD clinical research in accordance with regulatory requirements.

#### Data workflows

DOIs are processed using prespecified workflows. Workflows are defined using an ordered set of elementary actions: insert, edit, delete, annotate, link, copy, and execute. More complex workflows support conditional logic and parallel or asynchronous actions. The state of each DOI is updated and logged as it makes its way through a given workflow. The execute action merits special mention, as it may be used to define workflows that incorporate functionality conducted by external systems. This capability allows the ATRI EDC system to coordinate complex study-specific activities across multiple systems.

### Core modules

#### Electronic remote data capture of eCRFs

The EDC module serves as the primary interface for remote study data collection and implements user role-, eCRF-, and site-based permissions. A novel user interface design supports fluid and efficient navigation, review, and input of participant records by seamlessly combining eCRF, query, and audit trail information.

#### EDC use by trial sites

**Figure 4.**
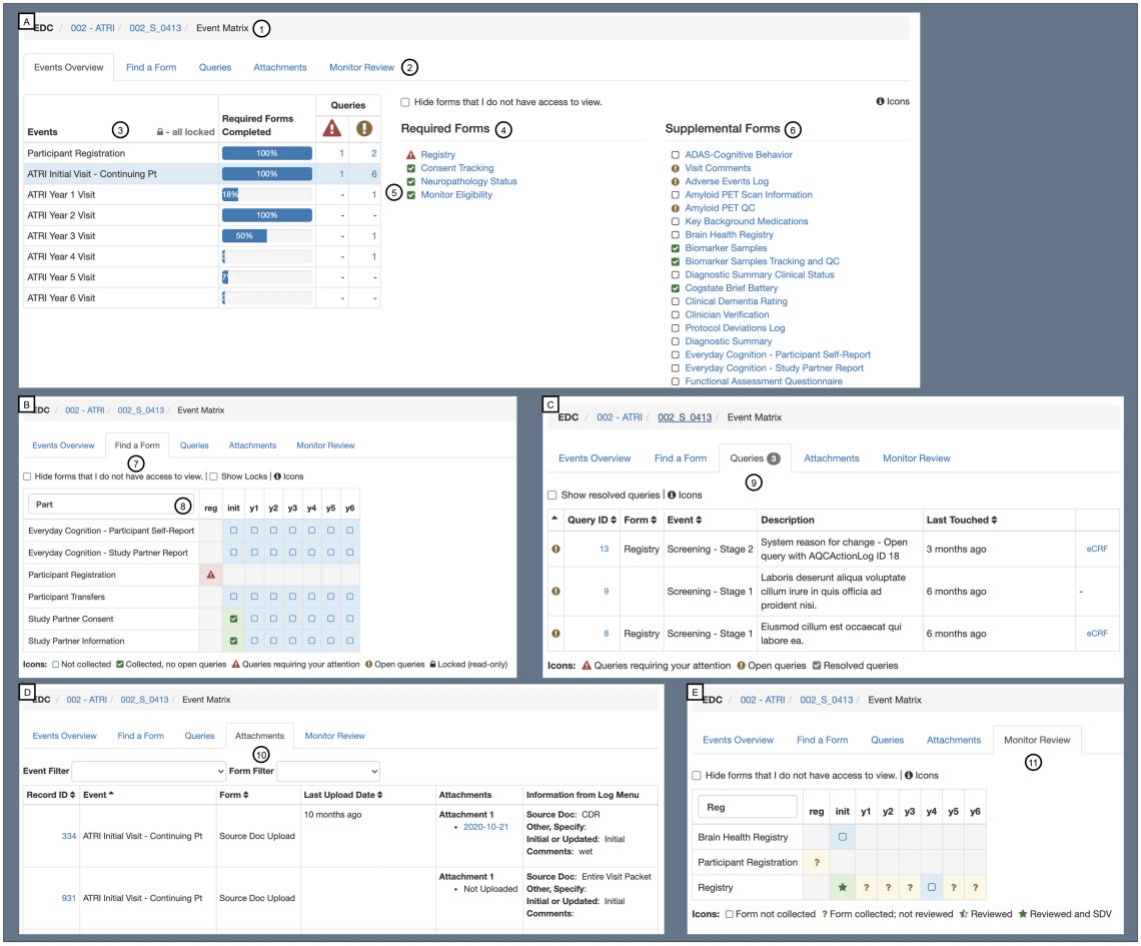
ATRI EDC Participant Event Matrix interface. This interface offers a unified overview of a participant’s study record by integrating information from multiple sources—eCRFs, queries, file uploads, data locking, clinical monitor review, and source document verification (SDV). Panel A demonstrates the “Events Overview” tab view, which includes the following features: (1) case navigation and orientation, (2) interface tab selector, (3) event dashboard with study event lock status, eCRF completion progress meter, and unresolved query counts, (4) list of required eCRFs, (5) eCRF completion status, and (6) list of supplemental eCRFs and completion status. Panel B demonstrates the “Find a Form” tab view, which includes the following features: (7) eCRF completion status per study event dashboard, and (8) eCRF name search. Panel C demonstrates the “Queries” tab view, which features the unresolved query dashboard (9). Panel D demonstrates the “Attachments” tab view, which features the file uploads dashboard (10). Panel E demonstrates the “Monitor Review” tab view, which features the eCRF review status per study event dashboard (11).

**Figure 5.**
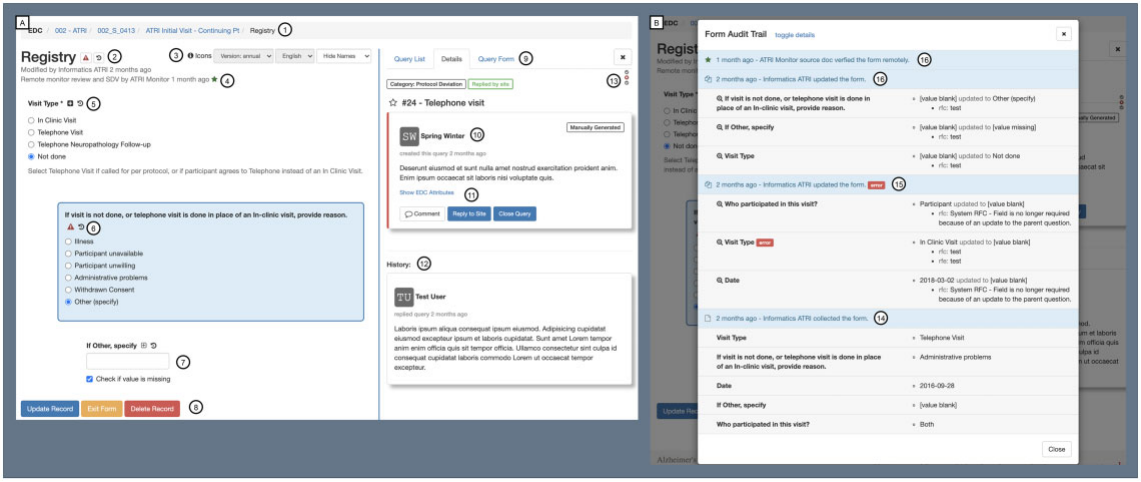
ATRI EDC integrated eCRF-Query-Audit Trail interface. This novel interface integrates multiple data sources—eCRF, source document verification (SDV), record locking, eCRF- and item-level queries, eCRF- and item-level audit trail, and eCRF version and revision information. It supports accurate and timely communication and collaboration between study teams and sites by offering a shared view of these data sources and a conversational interface for data query creation and resolution. Panel A demonstrates the primary eCRF interface, which includes the following features: (1) case navigation and orientation, (2) 1-click eCRF-level query and audit trail review, (3) eCRF version and language settings, (4) dates of last modification and SDV, (5) various input types with accompanying prompt, units, notes, and 1-click item-level query and audit trail review, (6) real-time item-level error feedback with query integration, (7) item indenting, missing data check box, and skip patterns, (8) color-coded action buttons, (9) integrated query listing, details, and creation, (10) conversational query interface, (11) query comments, response, and closure, (12) query conversation history, and (13) field navigation, orientation, and query status. Panel B demonstrates the integrated Audit Trail interface, which includes the following features: (14) summary and detailed view of eCRF insert, (15) summary and detailed view of eCRF update with item-level change summary and query status, (16) eCRF update with resolved query status, and (17) SDV summary.

Site personnel use the EDC module to collect participant eCRF data, upload source documents, and submit neuroimaging studies at predefined study event timepoints. The Participant Event Matrix interface displays the status of all captured, queried, and missing data for participants at their site (Figure 4). Newly captured data are validated against a battery of edit checks and quality control rules; errors are immediately reported to the user for correction (Figure 5). Unresolved errors automatically lead to the generation of queries to facilitate tracking and resolution. All transactions on study data are recorded in the audit trail and may also trigger notifications to the study team or asynchronous data processing pipelines.

#### EDC use by study teams

Study teams use the EDC module to conduct data management and monitoring activities across all study sites. The EDC module supports the creation and resolution of item- and eCRF-level data queries, data and audit trail review, data locking, and source document verification.

#### Shared use of the EDC module facilitates communication and collaboration

The shared view of a participant’s comprehensive data record, which integrates eCRF, query, and audit trail information, facilitates efficient and effective communication and collaboration between the study team and trial sites.

#### Metadata-driven EDC and eCRF configuration

Metadata drives the EDC module’s functionality and features. In the case of an eCRF, this includes item names, prompts, edit checks, quality rules, and skip patterns (Table 2). New metadata is validated using a comprehensive set of quality and consistency checks. Once validated, the new metadata updates the EDC module’s web-based views, functionality, and eCRF configurations. These configurations are managed by the system’s metadata model, using a version and revision control scheme, and applied prospectively, maintaining the integrity of previously captured data. This metadata-driven approach allows study teams to implement intrastudy protocol amendments in a well-controlled manner.

**Table 2.** ATRI EDC eCRF configuration metadata dictionary

Class label	Attribute label	MLS	Description
CRF metadata	CRF_NAME	—	Short name of the eCRF.
	CRF_LABEL	—	Long name of the eCRF.
	ALLOW_MULTIPLE_ENTRY	—	Allow multiple records of an eCRF to be collected at a single study visit.
	IS_CROSS_EVENTS	—	Allow multi-entry eCRFs to span multiple study visits; used for concurrent medications and adverse event logs.
	ALLOW_CHANGE_VERSION	—	Allow users to choose which version of an eCRF they will collect, otherwise, eCRF versions are selected by the system.
	LOG_MENU_FIELD	—	Specifies the set of fields and labels that will be presented in the multi-entry eCRFs log view.
Version metadata	V_VERSION	—	eCRF version name; multiple versions of the same eCRF can be defined.
Revision metadata	FORM_SUBTITLE	Yes	eCRF subtitle.
	FORM_INSTRUCTION	Yes	eCRF instructions, below subtitle.
Fieldset metadata	TITLE	Yes	Field section title.
	DESCRIPTION	Yes	Field section description.
Field metadata	FIELD_NAME	—	Field short name.
	QUESTION	Yes	Field prompt.
	ORDER	—	Field order number.
	UNITS	Yes	Field units (not required).
	NOTES	Yes	Filed notes (not required); supports Markdown.
	WIDGET	—	Field input type: select; multiCheckbox; input; textarea; radio; radioHorizontal; pin; datepickerStrict; datepicker; timepicker24. System allows key/label pairs to be specified for input types that support multiple choice options.
	TYPE	—	Field type: (T) Text; (N) Numeric; (D) Date.
	VALUE	—	Range for a numeric field: *[min value]…[max value]:[step]*
	REQUIRED	—	Indicate if the field is required.
	ALLOW_MISSING	—	Indicate if the field is allowed to be collected as “missing”; a “missing” checkbox input will be appended to this field in the eCRF.
	LENGTH	—	Length of the field: *[number, decimal]*
	PHI	—	Indicate if the field may contain PHI/PII; will be masked or removed from data exports by default.
	INDENT	—	Field indentation from the left margin of the eCRF.
	SUBQUESTION	—	Specify conditional field visibility rules and skip patterns.
	DEFAULT_VALUE	—	Specify a default value for the field.
	DOUBLE_ENTRY	—	Activate field-level double entry.
File metadata	NAME	—	File attachment: File name.
	LABEL	Yes	File attachment: File label.
	RESTRICTIONS	—	File attachment: File extension restriction. By default, no restrictions are applied.

*Note:* Attribute templates are indicated in italics.

*Abbreviation*: MLS: multi-language support.

#### Annotated eCRFs

The annotated eCRFs module offers an additional mechanism to support collaborative study design and development. This module allows study teams to generate a highly detailed, printable representation of each version and revision of an eCRF annotated with configuration information such as item name, prompt, coding, edit checks, and skip patterns (Table 2). These documents facilitate effective and efficient multistakeholder eCRF design, review, and validation processes.

#### Query management

The Query module is designed to promote transparency and shared accountability between study teams and sites for the timely resolution of data quality issues. It is capable of functioning as a standalone module or embedded within other modules. It implements a novel conversational user interface that supports query-related communication and collaboration. These interactions are recorded in the system’s audit trail and can be easily referenced and analyzed to identify personnel training issues or eCRF improvements. Query status changes trigger role-based alerts and notifications. Queries can also be annotated and categorized to support further analysis and reporting. A suite of reports provides query performance metrics that study teams can use to manage data quality throughout a study.

#### Audit trail

Consistent with regulatory requirements, every transaction (insert, update, delete, and upload) performed on a study-CDMS’s study metadata and data is recorded in the system’s audit trail. The Audit trail module integrates information from multiple sources (metadata, item data, reason for change [RFC], file version, query data, data locks, source document verification) to present a comprehensive view of the factors associated with study data changes over time.

#### Document management

The Document Repository module provides a scalable, version-controlled mechanism to manage file-based data such as documents, videos, spreadsheets. It allows users to access, preview, upload, download, and annotate file-based data. The module implements a file-system interface and supports the organization of files by topic, a construct like a single-level file-system folder. Users may subscribe to receive notifications when new files are uploaded to or deleted from a specific topic. User preview and download activity in this module is recorded in the module’s usage log.

#### Topic types

The Document Repository module supports multiple topic types, each with its associated metadata, configurations, and workflows. Standard topics offer file version control, annotations, and RFC features. Site topics have identical functionality, but automatically create site-specific sub-topics as new trial sites to the study-CDMS. In addition to role- and topic-based permissions, these site-specific sub-topics implement site-based permissions. Additional topic types are available to support data archival (read-only) and large data transfers (read-write).

#### Data transfers

The Document Repository module’s functionality—access control, version control, file retention, annotations, RFC, notifications, and logging—makes it a robust mechanism to facilitate inbound and outbound data transfers. To further improve support for this use case, file naming and content validation rules can be configured and enforced at the point of upload.

#### Data export

The Data Export module provides a standardized mechanism to export study metadata and data. The module supports export to multiple formats: comma-separated (2-dimensional) and JSON (multi-dimensional). As of August 2021, export to additional formats, such as PDF, R, and CDISC ODM, is under development.

#### Data lake

As previously noted, AD/ADRD studies collect large heterogeneous databases from multiple sources. Loading external data into the study-CDMS using traditional ETL methods is labor-intensive and fragile. At scale, providing users with real-time exports of study data from a study-CDMS is inefficient. One approach to address these issues involves importing all study data regularly to a semi-structured data repository, also referred to as a “data lake.”^26^ This approach bypasses the need to upload data into a structured data store, such as a relational database or data warehouse, while still providing indexing and standardization to improve discoverability and usability. The Document Repository module can be configured to serve as an interface to a study-specific data lake to facilitate user access.

#### Standard reports

The Reports module provides a standard suite of reports that are available on all CDMS instances. These reports summarize study operations, data inventories, data quality, and data flows.

#### Additional modules

**Figure 6.**
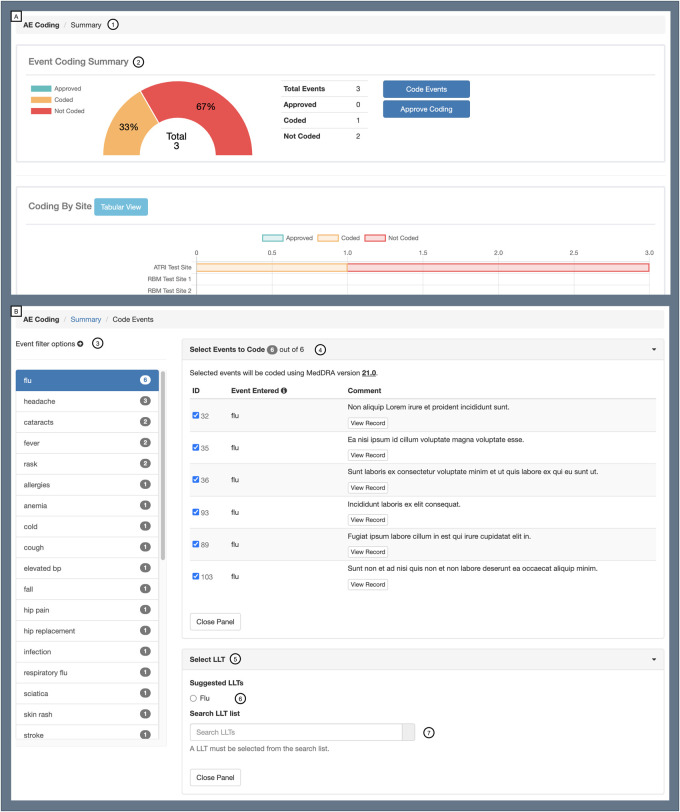
ATRI EDC additional modules example: Adverse Event Coding module. This module supports the coding of adverse event (AE) data. It leverages a code suggestion feature powered by a text classification API that combines natural language processing (NLP) and machine learning methods.^30^ Panel A demonstrates the Event Coding Summary view, which includes the following features: (1) navigation and orientation and (2) event coding summary dashboard. Panel B demonstrates the Event Coding interface, which includes the following features: (3) event filtering and grouping, (4) event coding selection, and (5) event code suggestion and search. LLT: MedDRA lowest level term.

The ATRI EDC system provides several additional modules that support the effective and efficient conduct of AD/ADRD clinical trials (Figure 6). These modules include randomization, medical image management, adverse event coding, safety reporting, and clinical monitor visit reporting. A complete description of these modules is beyond the scope of this article; detailed descriptions are planned for future articles.

#### Integration with external systems

The ATRI EDC system provides a comprehensive set of application programming interfaces (APIs) to support integration with external systems. Examples of these integrations include automating inbound and outbound data transfers with sponsors and vendors, ordering the shipment of investigational product at the point of participant randomization, and registering study participants in an external image management system.

## RESULTS

As of August 2021, the ATRI EDC system has supported the conduct of 10 AD/ADRD multicenter clinical studies (Table 3). Each study database was developed collaboratively by ATRI study teams in 3–6 months. Table 4 summarizes the modules and integrations with external systems that support each study. To date, the ATRI EDC has been used to capture data for 4596 study participants from trial sites in Asia, Australia, Europe, and North America. For these participants, more than 330 000 eCRFs have been collected. Among these studies, the 3 cases described below illustrate the ATRI EDC system’s flexibility and range of capabilities.

**Table 3.** Multicenter AD/ADRD clinical studies supported by the ATRI EDC system (as of August 3, 2021)

Study	Study type	Start year	Users	Sites	Participants screened	eCRFs		Queries		Document repository		Image studies
						N	Transactions	N	Rate (%)	Files	File versions	
ABC-DS	OC	2021	22	8	—	—	—	—	NAN	19	19	—
ADNI3	OC	2016	836	63	1802	136 876	152 773	11 644	8.5	1217	135 797	—
AHEAD3-45	RCT	2020	1065	108	1147	63 200	69 047	9275	14.7	4161	14 333	449
COGRX	RCT	2022	—	—	—	—	—	—	—	—	—	—
LEADS	OC	2018	311	19	415	28 843	34 497	4919	17.1	539	11 721	—
LIBBY	RCT	2022	12	—	—	—	—	—	—	—	—	—
MIND	RCT	2017	443	51	623	61 656	72 588	7361	11.9	446	34 040	—
NiAD	OC	2016	102	5	248	20 623	24 391	2601	12.6	11	4186	—
TRC-PAD	OC	2019	598	51	360	26 592	28 545	1086	4.1	486	13 740	252
TRC-DS	OC	2021	149	17	1	26	27	1	3.8	410	419	2
Total			3526	322	4596	337 816	381 868	36 887		7289	214 255	703

*Note:* Some sites participate in multiple studies. AD/ADRD RCTs typically experience 50%-80% screen failure rates; participant enrollment is ongoing in all studies. Two new RCTs, COGRX and LIBBY, are currently under development and scheduled to start in 2022.

Abbreviations: ABC-DS: Alzheimer Biomarker Consortium—Down Syndrome; ADNI3: Alzheimer’s Disease Neuroimaging Initiative 3 (ADNI3) Protocol; AHEAD3-45: AHEAD 3-45 Study: A Study to Evaluate Efficacy and Safety of Treatment With Lecanemab in Participants With Preclinical Alzheimer’s Disease and Elevated Amyloid and Also in Participants With Early Preclinical Alzheimer’s Disease and Intermediate Amyloid; COGRX: Randomized Double Blind, Placebo Controlled, Parallel Group Trial to Evaluate the Safety and Efficacy of CT1812 in Early Alzheimer’s Disease over 18 Months; LEADS: Longitudinal Early-onset Alzheimer’s Disease Study Protocol; LIBBY: Life’s end Benefits of CannaBidol and TetrahYdrocannabinol (LiBBY) Trial; MIND: Memory Improvement Through Nicotine Dosing (MIND) Study; NIAD: Neurodegeneration in Aging Down Syndrome (NiAD): A Longitudinal Study of Cognition and Biomarkers of Alzheimer’s Disease; TRC-PAD: TRC-PAD Program: In-Clinic Trial-Ready Cohort; TRC-DS: Trial-Ready Cohort-Down Syndrome; OC: observational cohort; RCT: randomized clinical trial.

**Table 4.** ATRI EDC modules and external integrations supporting AD/ADRD studies (as of August 3, 2021)

Study	Study type	Standard modules	Clinical monitor visit reporting	AE coding	Image management	Safety reporting	External integrations
ABC-DS	OC	X					Imaging
ADNI3	OC	X		X			Imaging, RBM, Labs
AHEAD3-45	RCT	X	P		X		IRT, ECOA, EPRO, RBM, Labs, ECG
COGRX	RCT	X	P	X	X	P	IRT, RBM, Labs
LEADS	OC	X		X			
LIBBY	RCT	X	P	X		P	IRT, RBM, Labs
MIND	RCT	X	X	X		X	IRT, RBM, Labs
NiAD	OC	X					
TRC-PAD	OC	X	P	X	X	P	RBM, Labs
TRC-DS	OC	X	P	X	X	P	RBM, Labs

*Note:* Standard modules are used in all studies and include eCRFs, Annotated CRFs, Query Management, Audit Trail, Document Management, Data Export, Standard Reporting, and Data Lake.

*Abbreviations*: ABC-DS: Alzheimer Biomarker Consortium—Down Syndrome; ADNI3: Alzheimer’s Disease Neuroimaging Initiative 3 (ADNI3) Protocol; AHEAD3-45: AHEAD 3-45 Study: A Study to Evaluate Efficacy and Safety of Treatment With Lecanemab in Participants With Preclinical Alzheimer’s Disease and Elevated Amyloid and Also in Participants With Early Preclinical Alzheimer’s Disease and Intermediate Amyloid; COGRX: Randomized Double Blind, Placebo Controlled, Parallel Group Trial to Evaluate the Safety and Efficacy of CT1812 in Early Alzheimer’s Disease over 18 Months; LEADS: Longitudinal Early-onset Alzheimer’s Disease Study Protocol; LIBBY: Life’s end Benefits of CannaBidol and TetrahYdrocannabinol (LiBBY) Trial; MIND: Memory Improvement Through Nicotine Dosing (MIND) Study; NIAD: Neurodegeneration in Aging Down Syndrome (NiAD): A Longitudinal Study of Cognition and Biomarkers of Alzheimer’s Disease; TRC-PAD: TRC-PAD Program: In-Clinic Trial-Ready Cohort; TRC-DS: Trial-Ready Cohort-Down Syndrome; OC: observational cohort; RCT: randomized clinical trial; P: module activation planned; X: module active; ECG: electrocardiogram; ECOA: electronic clinical outcome assessment; EPRO: electronic patient-reported outcomes; IRT: interactive response technology for randomization and study supplies; RBM: risk-based monitoring.

### Case: ADNI 3 study

The Alzheimer’s Disease Neuroimaging Initiative (ClinicalTrials.gov ID: NCT02854033) (http://adni.loni.usc.edu) (Table 3),^27^ a large multicenter prospective observational cohort study that seeks to enroll 2000 participants across various AD diagnostic groups (Cognitively Normal, Mild Cognitive Impairment, and Mild AD Dementia). The study is funded by a public–private partnership between the NIH and several private companies. In this study, the ATRI EDC system serves several purposes: (1) the EDC, Query, and Audit Trail modules are used to collect and manage the clinical database; (2) the Document Repository module supports study document management, inbound data transfers from multiple external biomarker labs, and large data exports to support analysis and statistical reporting; (3) the AE Coding module supports coding of adverse events; and (4) the system’s APIs integrate the study-CDMS with a data-sharing platform hosted by the USC Laboratory of Neuro Imaging (https://www.loni.usc.edu).

### Case: TRC-PAD study

The Trial Ready Cohort for the Prevention of Alzheimer’s Dementia (TRC-PAD) study (ClinicalTrials.gov ID: NCT04004767) (https://trcpad.org) (Table 3),^9^ a large multicenter prospective observational cohort study that seeks to enroll 2000 participants with increased risk of memory loss caused by AD. The study is funded by the NIH. In this study, the ATRI EDC system serves several purposes: (1) the EDC, Query, and Audit Trail modules are used to collect and manage the clinical database; (2) the Document Repository module supports study document management, inbound data transfers from multiple external biomarker labs, and large data exports to support analysis and statistical reporting; (3) the Imaging module supports the acquisition and management of PET neuroimaging studies; (4) the AE Coding module supports coding of adverse events; and (5) the system’s APIs integrate the study-CDMS with the TRC-PAD Informatics Platform and the APT Webstudy (https://www.aptwebstudy.org).^28^

### Case: AHEAD3-45 study

The AHEAD3-45 study (ClinicalTrials.gov ID: NCT04468659) (https://www.aheadstudy.org) (Table 3), a large multicenter double-blinded randomized clinical trial (RCT) that seeks to enroll 1400 participants to determine the efficacy of treatment with Lecanamab (BAN2401) relative to placebo in 2 diagnostic groups (Preclinical AD and Early Preclinical AD). The study is funded by a public-private partnership between the NIH and Eisai, Inc. The study is conducted via a global network of 108 sites in Asia, Australia, Europe, and North America. In this study, the ATRI EDC system serves several purposes: (1) the EDC, Query, and Audit Trail modules are used to collect and manage the clinical database; (2) the Document Repository module supports study document management, inbound and outbound data transfers with multiple external biomarker labs and vendors (ePRO, eCOA, IRT, RBM), and large data exports to support analysis and statistical reporting; (3) the Imaging module supports the acquisition and management of MRI neuroimaging studies; and (4) the system’s APIs integrate the study-CDMS with external pharmacovigilance, safety monitoring, and reporting systems.

## DISCUSSION

### Evolving requirements and technological innovation

The ATRI EDC system was developed to address the complex and demanding set of requirements of AD/ADRD clinical trials. As described in previous sections, the system’s flexibility and capabilities in supporting a wide array of complex study designs and operational implementations confirm that these requirements have been met. However, the pace of innovation in AD/ADRD clinical research continues to accelerate, requiring further development of the system. In parallel, rapid shifts in technology and computing offer opportunities to address existing and emerging study requirements using novel methods. Incorporating capabilities based on machine learning, microservices, and serverless computing into the ATRI EDC system that will increase data quality, improve productivity, and reduce the burden on study teams and sites are now being planned.

### Open science

Consistent with the principles of open science, the ATRI EDC system is intended to be shared with the scientific community. Interested investigators can obtain system source code and documentation for evaluation and use via a technology transfer agreement by submitting a request via email to: softwarelicensing@atrihub.io. The ATRI Informatics team hosts regular community-based webinars with investigators and their study teams to discuss the system roadmap, preview updates, and gather feedback. As an example, investigators from the University of Tokyo are using the ATRI EDC to support the J-TRC study,^29^ a multicenter prospective observational cohort study in preclinical and prodromal AD.

### Looking beyond AD/ADRD clinical studies

More generally, the ATRI EDC system has several advantages to offer other groups that are coordinating trials. Its flexibility and range of capabilities make it a suitable option for studies in other fields of medical research.

### Limitations

The ATRI EDC system has several limitations that must be acknowledged. First, the system was designed to address the requirements of large multicenter clinical studies, where the costs of commercial options are substantial and the capabilities of academic or community-based systems are incomplete. The advantages of this design, however, are not generalizable to all study types. A small single-center study, for example, may be better served by an academic or community-based EDC solution, which is simpler to use and more cost-effective. To address these limitations, we plan to develop a new “quick start” module with a simplified user interface that will guide study teams through the study configuration process in a stepwise manner, using a series of prompts to select appropriate system settings. We also plan to enhance our user and system documentation and training materials.

Second, despite efforts toward simplification, the technical expertise and training necessary to effectively administrate the system are significant and may represent a barrier to further adoption by other research organizations and teams. This may slow community-building efforts and innovation. To address this limitation, we plan to offer a containerized version of the system that will reduce the administrative requirements and facilitate hosting in a wider array of computational environments.

Finally, maintaining strict isolation of the system’s application code and data is achieved at the expense of higher resource utilization and storage density. This approach is less cost-effective and has a higher environmental impact.

## CONCLUSION

In conclusion, AD/ADRD clinical trials present a complex set of scientific, operational, and regulatory challenges that require innovative technology and team-based science. ATRI designs and conducts large multicenter AD/ADRD clinical studies across a global network of sites by building multidisciplinary study teams of clinical trialists and leveraging the increasing functionality and capabilities of the ATRI EDC remote data capture system. The combination of human expertise and novel technologies facilitates ATRI’s mission to conduct rigorous and efficient therapeutic AD/ADRD trials.

## AUTHOR CONTRIBUTIONS

GJM conceived and led the project. GJM, SB, HQ, and JS designed and implemented the project. GJM, SB, HQ, and JS participated equally in the writing of the manuscript. PSA provided scientific and medical expertise. All authors provided feedback and reviewed and approved the final version of the manuscript.
