# Corrigendum to: ATRI EDC: a novel cloud-native remote data capture system for large multicenter Alzheimer’s disease and Alzheimer’s disease-related dementias clinical trials

**DOI:** 10.1093/jamiaopen/ooac008

**Published:** 2022-02-24

**Authors:** Gustavo A Jimenez-Maggiora, Stefania Bruschi, Hongmei Qiu, Jia-Shing So, Paul S Aisen

*JAMIA Open*, Volume 5, Issue 1, April 2022, ooab119,https://doi.org/10.1093/jamiaopen/ooab119

In the originally published version of this manuscript, the title was incorrect.

The incorrect title read:

ATRI EDC: a novel cloud-native remote data capture system for large multicenter Alzheimer’s disease and Alzheimer’s disease-related dementias clinical trails

This has now been corrected to:

ATRI EDC: a novel cloud-native remote data capture system for large multicenter Alzheimer’s disease and Alzheimer’s disease-related dementias clinical trials

